# Real-Time Pipe and Valve Characterisation and Mapping for Autonomous Underwater Intervention Tasks

**DOI:** 10.3390/s22218141

**Published:** 2022-10-24

**Authors:** Miguel Martin-Abadal, Gabriel Oliver-Codina, Yolanda Gonzalez-Cid

**Affiliations:** Department of Mathematics and Computer Science, University of the Balearic Islands, 07122 Palma, Spain

**Keywords:** autonomous intervention, underwater perception, deep learning, point cloud segmentation, pipeline characterisation, pipeline mapping, real-time

## Abstract

Nowadays, more frequently, it is necessary to perform underwater operations such as surveying an area or inspecting and intervening on industrial infrastructures such as offshore oil and gas rigs or pipeline networks. Recently, the use of Autonomous Underwater Vehicles (AUV) has grown as a way to automate these tasks, reducing risks and execution time. One of the used sensing modalities is vision, providing RGB high-quality information in the mid to low range, making it appropriate for manipulation or detail inspection tasks. This work presents the use of a deep neural network to perform pixel-wise 3D segmentation of pipes and valves on underwater point clouds generated using a stereo pair of cameras. In addition, two novel algorithms are built to extract information from the detected instances, providing pipe vectors, gripping points, the position of structural elements such as elbows or connections, and valve type and orientation. The information extracted on spatially referenced point clouds can be unified to form an information map of an inspected area. Results show outstanding performance on the network segmentation task, achieving a mean *F1-score* value of 88.0% at a pixel-wise level and of 95.3% at an instance level. The information extraction algorithm also showcased excellent metrics when extracting information from pipe instances and their structural elements and good enough metrics when extracting data from valves. Finally, the neural network and information algorithms are implemented on an AUV and executed in real-time, validating that the output information stream frame rate of 0.72 fps is high enough to perform manipulation tasks and to ensure full seabed coverage during inspection tasks. The used dataset, along with a trained model and the information algorithms, are provided to the scientific community.

## 1. Introduction

The need for conducting underwater intervention tasks has grown significantly in recent decades. Often it is necessary to perform underwater operations in different fields such as archaeology, biology, rescue and recovery or industry that include not only inspection but also interaction with the environment. One of the most relevant cases is the manipulation tasks performed on offshore oil and gas rigs or pipeline networks [1,2,3,4,5].

In the past, the aforementioned tasks were mostly carried out, manually, by scuba divers. Nonetheless, conducting these missions in a hard-to-reach scenario such as open waters tends to be slow, dangerous and resource-consuming. Recently, Remotely Operated Vehicles (ROVs) equipped with diverse sensing systems and manipulators have been used to access deeper and more complex underwater scenarios, allowing the elimination of some of the drawbacks of human intervention.

However, ROVs still presented downsides such as its hard and error-prone piloting due to complex water dynamics, requiring trained operators; or the need for a support vessel, leading to expensive operational costs. To ease these drawbacks, there has been increasing research towards intervention Autonomous Underwater Vehicles (AUVs) [6,7,8] and Underwater Vehicle Manipulator Systems (UVMS) [9,10].

Other challenges faced in underwater environments are presented regarding sensing in general and object perception in particular. Underwater sensing presents several challenges such as distortion in signals, light absorption and scattering, water turbidity changes or depth-depending colour distortion.

Intervention ROVs and AUVs are often equipped with a variety of sensing systems. When operating in unknown underwater environments, sonar systems are usually preferred as they are able to obtain bathymetric maps of large areas in a short time. Even though sonar is mostly used to provide general information about the environment or used in a first-stage approach to the area of interest, it has also been used to perform object detection by itself. Nonetheless, the preferred sensing modalities to obtain detailed, short-distance information with higher resolution are laser and video. These modalities are often used during the approach, object recognition and intervention phases. The use of the presented sensing systems towards object detection and, specifically, pipe and valve recognition is reviewed in Section 2.1.

To execute manipulation tasks, the sensing systems of a ROV or AUV must be able to provide enough information to perform accurate and robust scene understanding, including object detection, target recognition and pose estimation, under different experimental conditions.

This paper is a continuation of our previous work [11] where we proposed a deep learning-based approach to perform a pixel-wise segmentation of underwater pipes and valves from 3D RGB point cloud information.

In this paper, we make use of an improved evolution of the deep neural network used in our previous work to perform pixel-wise 3D segmentation. Additionally, we implement object detection over the segmented pixels, grouping them and being able to detect diverse pipe and valve instances in a point cloud. We develop an algorithm to extract information from the detected instances providing pipe vectors, gripping points, structural elements such as elbows or connections, and valve type (2-way or 3-way) and orientation. Furthermore, if the point clouds are spatially referenced, their information can be unified, forming an information map of an inspected area. Finally, the 3D segmentation, along with the information extraction and mapping algorithms, are executed online on an AUV, performing real-time underwater pipe and valve recognition, characterisation and mapping for inspection and manipulation tasks.

The remainder of this paper is structured as follows: Section 2 reviews the related work on underwater perception and pipe and valve identification, and highlights the main contributions of this work. Section 3 describes the methodology and materials adopted in this study. The experimental results are presented and discussed in Section 4. Section 5 details the network and information algorithms online implementation. Finally, Section 6 outlines the main conclusions and future work.

## 2. Related Work and Contributions

### 2.1. State of the Art

This section reviews the use of diverse sensing systems for underwater inspection, object detection and, specifically, pipe and valve recognition. The three most used sensing systems in underwater environments are sonar, laser and vision.

Sonar sensing is the preferred method when working in large, unknown environments, providing broad information in a quick manner [12,13]. It has also been used for object localisation in underwater scenarios [14,15]. Object detection deep learning techniques have been applied to underwater sonar imaging for diverse applications such as the detection of human bodies [16] or war mines [17]. Some of the drawbacks presented by sonar imaging are the noisy nature of the images, which generates texture information losses, and the fact that it is not able to capture colour information, which is useful in object recognition tasks.

Underwater laser scans can provide high-resolution 3D data that can be used for environment inspection and object recognition. Some studies on underwater pipeline detection include the works of Palomer et al. [18] where a laser scanner is integrated on an AUV for object detection and manipulation, or the works of Himri et al. [19,20] and Villacrosa et al. [21], where a recognition and pose estimation pipeline based on point cloud matching is built. As for downsides, laser systems tend to have a very high initial cost, are affected by light transmission problems and cannot provide colour information.

Vision is one of the most complete and used perception modalities in robotics and object recognition tasks thanks to its accessibility, ease of use and the fact that produces RGB high-quality information. It also has disadvantages as the obtained images are affected by light transmission problems, colouring distortions or environmental factors such as water turbidity. Nonetheless, some of these weaknesses can be alleviated by adapting the acquisition system to the environmental conditions, adjusting the operation range, calibrating the cameras or colour correcting the obtained images.

In the past, traditional computer vision approaches have been used to detect and track multiple submerged objects such as artifacts [22,23,24,25], cables [26,27,28] or pipelines [28,29,30,31]. Some works rely on texture and shape descriptors [28,31], others on template matching [32,33] or use colour segmentation to find and process regions of interest in the images [25,34].

Other works use a combination of multiple sources of information, Kallasi et al. in [35] and Razzini et al. in [7,36] present traditional computer vision methods that combine texture, shape and colour information to detect underwater pipelines and project them into point clouds obtained from stereo vision. In these works, the point cloud information is not used to assist the pipe recognition process.

Rekik et al. [37] developed the first trainable system to detect underwater pipelines, extracting several features and using a Support Vector Machine to classify between positive and negative underwater pipe image samples. Convolutional Neural Networks were introduced by Nunes et al. [38] to classify diverse underwater objects, including a pipeline. None of these works determined the position of the object within the image, only a binary classification of the object’s presence was given.

Some studies introduced deep learning solutions applied to underwater computer vision, but are limited to the detection and pose estimation of 3D-printed objects [39] or living organisms such as fishes [40] or jellyfishes [41]. Few research studies involving pipelines are restricted to damage evaluation [42,43] or pipeline navigation from the inside [44]. Guerra et al. in [45] present one of the most advanced works on pipeline recognition using deep learning, where a drone equipped with a monocular camera is used to perform 2D detection of pipelines in industrial environments.

Therefore, with the exception of the later works of Himri et al. [20] and Villacrosa et al. [21], which will be later discussed in Section 4.1, the remaining works suffer from crucial drawbacks when tackling pipe and valve recognition for inspection and manipulation tasks. The most significant drawbacks from previous implementations, which are solved in our work, are listed below:

– Only recognising pipes, no valves, connections or elbows are detected.
– Not being able to detect multiple elements simultaneously, due to the nature of its data processing, only isolated objects can be detected.
– Not gathering information from the detected objects, such as pipe length, gripping points, orientation or valve type and position.
– Not being able, or no demonstration, of being able to be executed online on an inspecting or manipulating robot.

Finally, to the best knowledge of the authors, the only prior known research on underwater pipeline and valve 3D recognition using deep learning is our previous work presented in [11], upon which we build the research introduced in this paper.

### 2.2. Main Contributions

The main contributions of this paper are composed of:

1. Expansion of our novel point cloud dataset of underwater pipe and valve structures, adding point clouds obtained with a new pair of cameras mounted on an AUV. This dataset is used to train and test the selected deep neural network and information algorithms.
2. Development of novel algorithms to extract information from detected pipe and valve instances, providing data on pipe vectors, gripping points, structural elements such as elbows or connections, and valve type and orientation. The information from spatially referenced point clouds can be unified to create information maps of inspected areas.
3. Neural network and information algorithms validation by conducting underwater experiments where the point cloud segmentation, the information extraction algorithm and mapping algorithm are executed online in an AUV, performing real-time underwater pipe and valve recognition, characterisation and mapping for inspection and manipulation tasks.
4. The updated dataset (point clouds and corresponding ground truths) along with a trained model and the code of the algorithms used to perform the information extraction and mapping are provided to the scientific community in [46].

## 3. Methodology

This section presents an overview of the selected network; explains the acquisition, labelling and organisation of the data; details the information extraction and mapping algorithms; and exposes the validation process and evaluation metrics.

### 3.1. Deep Learning Network and Training Deatils

Even though most applications in the field work with 2D information, which some later project to the 3D space, we decided to use a 3D segmentation network using point clouds as input for diverse reasons. First of all, the introduction of depth data provides extra information to work with, allowing us to extract more features, helping the segmentation. Secondly, as we extract information from the segmented point clouds for inspection and manipulation tasks, 3D positioning would be a must. Thus, it would not make sense to use 2D segmentation to avoid possible matching failures in the 3D point cloud generation if the extracted information could not be projected into a 3D space.

In this work, we select the Dynamic Graph Convolutional Neural Network (DGCNN) [47] to perform the pipe and valve 3D segmentation. This network is an evolution of the PointNet deep neural network [48] that we used in our previous work, surpassing its performance on several benchmark datasets [47]. Like its predecessor, this network has a unified architecture that allows it to perform multiple tasks, ranging from object classification and part segmentation to scene semantic segmentation.

The novelty of the DGCNN architecture is the introduction of the named EdgeConv modules, which can be integrated into existing deep learning models such as PointNet. These modules capture local geometric structure information by generating edge features that describe relations between a point and its neighbours while being invariant to input permutations. Since proximity in the feature space differs from proximity in the input point cloud, the set of neighbours of a point changes from layer to layer. This results in a network graph that is updated after each network layer, leading to non-local diffusion of information over the whole point cloud. This allows the EdgeConv modules to capture global shape information.

Furthermore, to make the prediction invariant to the point cloud geometric transformations, all input sets are aligned to a canonical space before feature extraction. To achieve this, a 3 × 3 matrix is applied, obtained from a tensor concatenating the coordinates of each point and the coordinate difference between its neighbours.

The DGCNN architecture takes point clouds as input and outputs a class label for each point. While training the network, it is also fed with ground truth labels, indicating the real class of each point from the point cloud. The labelling process is further detailed in Section 3.2.2.

As the original DGCNN implementation, we use a softmax cross-entropy loss along with an Adam optimiser. The decay rate for batch normalisation starts with 0.5 and is gradually increased to 0.99. In addition, we apply a dropout with a keep ratio of 0.7 on the last fully connected layer, before class score prediction.

Other hyperparameters are selected based on the experiments conducted in our previous work [11]. For the training, we use stochastic gradient descent with a learning rate of 0.001, a batch size of 16; block and stride distances of 1 m; and, finally, a maximum number of allowed points per block of 128. These hyperparameters have proven to offer very good metrics in terms of point cloud segmentation while greatly reducing inference time, a key factor in the online execution of this network in an AUV to perform real-time inspection and manipulation tasks. Details on the network and algorithms online execution in an AUV are given in Section 5.

**Figure 1:**
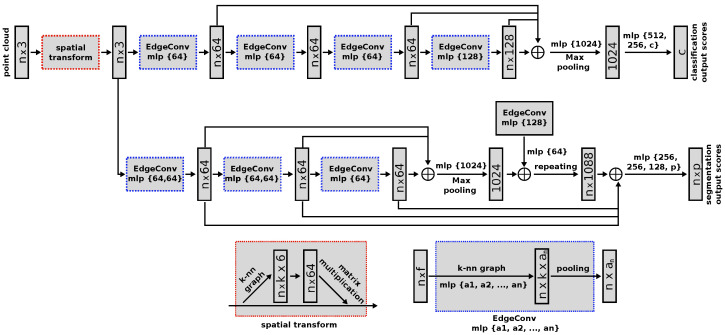
DGCNN architecture. Taken from [47], with permission from author Yue Wang, 2021.

To improve the network performance, we implement an early stopping strategy based on the work of L. Prechelt in [49], ensuring that the network training process stops when the divergence between validation and training losses is minimum. This technique allows us to obtain more general and broad training, avoiding overfitting. The DGCNN architecture is presented in Figure 1.

### 3.2. Data

This subsection explains the acquisition, labelling and management of the data used to train and test the deep neural network.

#### 3.2.1. Acquisition

The used point clouds come from two different sources. First, we reuse our previous dataset from [11], consisting of 192 underwater point clouds containing diverse pipe and valve structures and connections. This dataset was gathered by performing diverse surveys on an artificial pool with an Autonomous Surface Vehicle equipped with a Bumblebee2 Firewire stereo rig and using the Robot Operating System (ROS) middleware [50].

**Figure 2:**
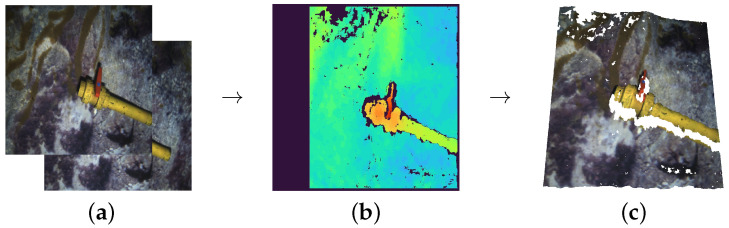
Data acquisition process: (**a**) Left and right stereo images from a calibrated stereo rig; (**b**) Disparity depth image obtained using ROS stereo processing [51]; (**c**) Point cloud generated by merging depth and colour information (Blank spaces correspond to areas where no matching between stereo images could be found or to covered areas).

The second source of point clouds is another stereo pair rig, composed of two Manta G283 cameras mounted on an AUV, gathering point clouds through ROS once again. We performed up to six immersions with the AUV to record different pipe structures and valve connections, two of them at an artificial pool, and the remaining four at different locations at the sea. The acquisition process is pictured in Figure 2.

#### 3.2.2. Ground Truth Labelling

**Figure 3:**
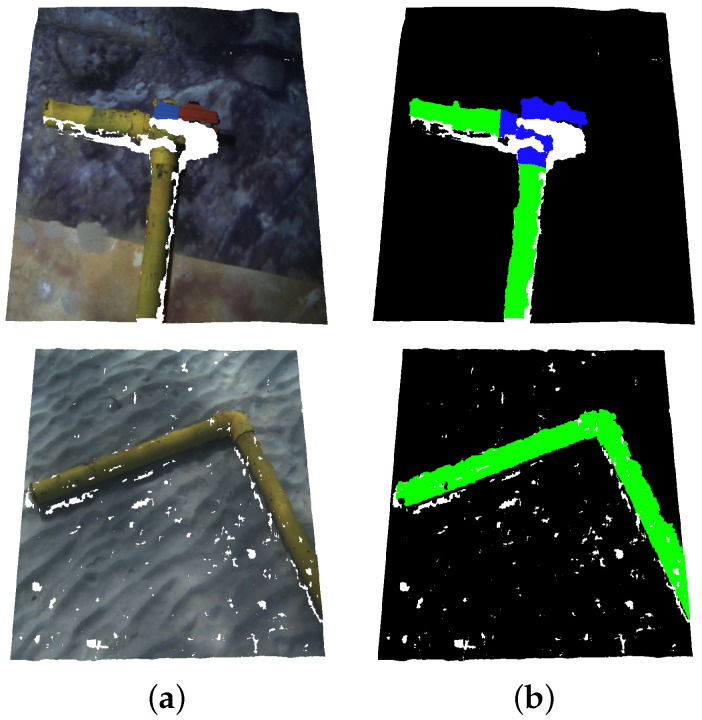
Point cloud labelling: (**a**) Original point cloud; (**b**) Ground truth annotations. Points corresponding to pipes, valves and background, are marked in green, blue and black, respectively.

In order to train and test the network, ground truth label maps are manually built from the obtained point clouds. The points corresponding to each class are marked with a different label. The studied classes and their RGB labels are: *Pipe* (Green: 0, 255, 0), *Valve* (Blue: 0, 0, 255) and *Background* (Black: 0, 0, 0). Figure 3 shows a couple of point clouds along with their corresponding ground truth label maps.

#### 3.2.3. Dataset Management

To configure our dataset, we gather the point clouds obtained from the two previously mentioned sources in Section 3.2.1. First, we take 192 point clouds from our previous dataset (from now on, referred to as set *S_ASV_*). Second, we extract point clouds from the AUV immersions. From the two pool immersions we extract 104 and 51 point clouds (from now on, referred to as sets *S_POOL-1/2_*, respectively). From the four sea immersions, we extract 45, 56, 36 and 30 point clouds (from now on referred as sets *S_SEA-1/2/3/4_*, respectively).

In order to build the dataset used for the training, validation and test, we decided to gather the point clouds from the sets *S_ASV_*, *S_POOL-1_* and *S_SEA-1/2_*, conforming a total of 397 point clouds along their corresponding ground truth label maps.

This dataset contains point clouds gathered using two different pairs of stereo cameras, in fresh and salt water, under different environmental conditions and showcasing a wide variety of pipe structures and valve connections over different backgrounds, such as a plastic lone, sand or rocks. This represents a broad spectrum of scenarios to assure robustness in the network training and reduce its overfitting. The dataset is split into a train-validation set (90% of the data, 357 point clouds) and a test set (10% of the data, 40 point clouds), which will be referred to as *T_BASE_*. Additionally, sets *S_POOL-2_* and *S_SEA-3_* are used to perform a secondary test, from now on referred as *T_EXTRA_*, containing a total of 87 point clouds. The point clouds from this test are from immersions whose data has not been used for the network training or validation, and contain different, unseen environmental conditions, pipe structures, valve connections and backgrounds. Hence, this test allows us to assess how well the network generalises its training to new data.

Finally, set *S_SEA-4_* contains point clouds gathered by the AUV navigating over a larger structure containing multiple pipes and valves. This set will be used to test the mapping algorithm presented in Section 3.3. This test set will be referred to as *T_MAP_*.

**Figure 4:**
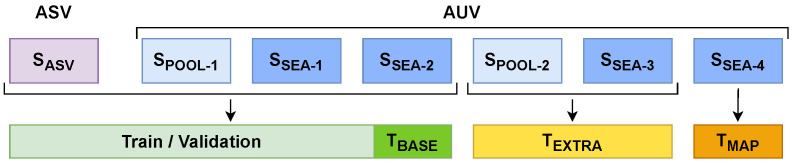
Dataset management.

**Figure 5:**
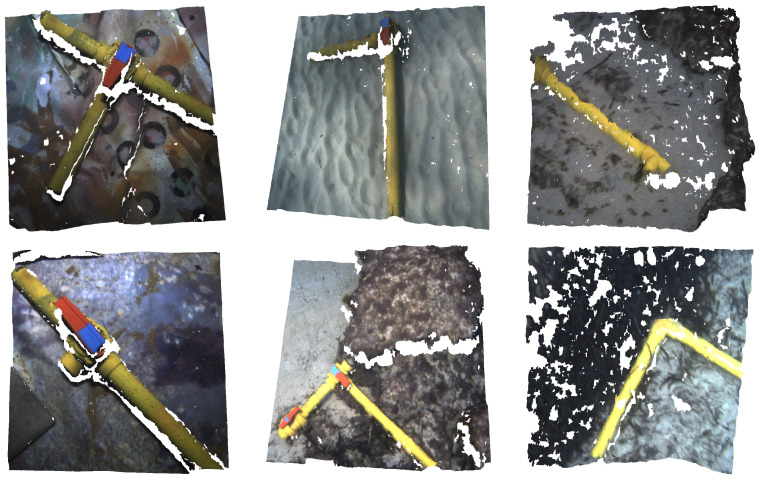
Point clouds from the dataset showcasing diverse pipe structures and valve connections over different backgrounds.

Figure 4 illustrates the dataset management, while in Figure 5 some examples of point clouds are shown.

### 3.3. Segmentation Understanding Algorithms

Once the deep neural network has processed a point cloud and generated its semantic segmentation, we need to further process this segmentation and extract information to use in inspection and manipulation tasks. To do that, we develop two algorithms. The first algorithm, referred to as Information Extraction Algorithm (IEA), takes the network output and extracts information such as the number of pipes and valves present in the point cloud, their position, orientation or even pipe connections and valve type (2-way or 3-way). The second algorithm, referred to as Information Unification Algorithm (IUA), unifies the information extracted from multiple localised point clouds taken on a studied area and generates a global information map. Next, both IEA and IUA algorithms are detailed.

#### 3.3.1. Information Extraction Algorithm

**Figure 6:**
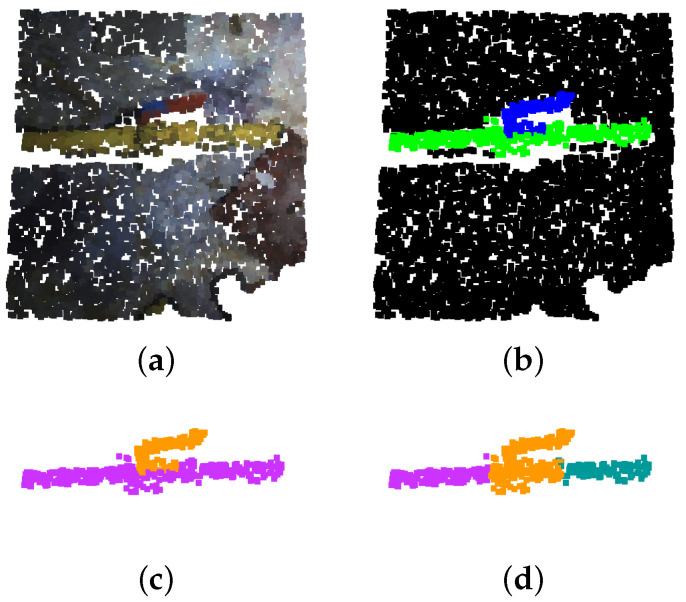
Point cloud clustering and valve reclassification. (**a**) Original point cloud. (**b**) Deep neural network segmentation. (**c**) Instance clustering. (**d**) Valve reclassification. Instances are represented following the purple, orange, teal and garnet colour sequence.

The starting point of this algorithm is the semantic segmentation outputted by the deep neural network. Its first step is to transform the pixel-wise segmentation into an instance-based one using a Density-Based Spatial Clustering of Applications with Noise (DBSCAN), clustering pixels of the same class that are closer than a distance threshold, clusters that do not contain enough points to be considered as instances are deleted. This way, the different pipe and valve instances present in a point cloud are detected. Additionally, when a cluster belonging to a valve instance is found, it is set to “steal” the points belonging to pipe instances that are within a determined radius of the valve instance central point. This is due to the fact that, as the main body of the valve is very similar to the actual pipes, it sometimes gets misclassified as a pipe and only the handle of the valve is correctly classified, this way, the body of the valve is reclassified. Figure 6 shows the instance clustering and valve reclassification on a segmented point cloud.

The following step of the algorithm is to extract information from the detected valve instances. First, the central point of the valve instances is calculated as the average XYZ coordinate values of all its belonging points, noting the valve position. Second, the algorithm performs a point cloud registration between each valve instance and five-point cloud models of 2-way and 3-way valves, obtaining the rotation matrix and model that provides a maximum registration score, inferring its pose and type. Using the valve registration data and its pre-known shape, a vector is generated to indicate each valve size and orientation.

Valve instances that do not reach a certain registration score threshold with any point cloud model are discarded. Consequently, the previously mentioned “stolen” points corresponding to discarded valve instances are returned to their corresponding original pipe instances.

**Figure 7:**
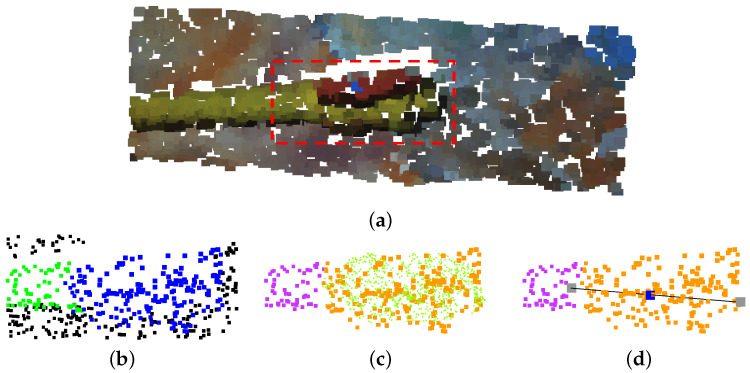
Valve information extraction process: (**a**) Original point cloud (area of interest highlighted in red square); (**b**) Deep neural network segmentation; (**c**) Instance clustering and best valve model registration (light green points); (**d**) Instance clustering along resulting valve central point (blue point) and vector (black line between the two gray points).

Figure 7 showcases the described valve information extraction process.

The next step is to extract information from the detected pipe instances. First, the point cloud instances are voxelized and flattened into a 2D matrix, where closing and opening morphology operations are performed to consolidate the instance as a unique object.

At this point, the skeleton of the instance is computed, obtaining a chain of linked matrix coordinates and depicting the pipe shape. Moreover, coordinates with up to three neighbours are marked as connection points between different pipes. Once the smaller chains are discarded, the remaining chains and connection points are reprojected into the original instance 3D points.

Then, the algorithm calculates the 3D vector between each chain point and its linked points, providing information about the chain curvature. From there, pipe elbows can be established on point sequences with greater curvature than a selected threshold. Moreover, these vectors provide information on the chain length, allowing us to locate the position of a determined percentage of pipe length, this information is very useful to provide grabbing points for pipe manipulation. Finally, a vector describing each straight portion of a chain is calculated between its first and last point, giving information on the corresponding pipe orientation and length.

**Figure 8:**
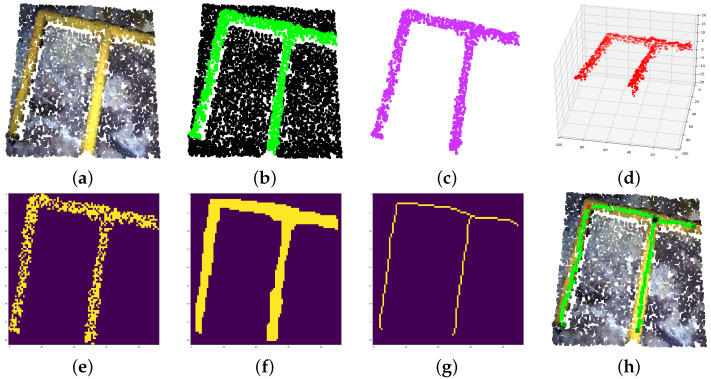
Pipe information extraction process. (**a**) Original point cloud. (**b**) Deep neural network segmentation. (**c**) Instance clustering. (**d**) 3D voxelization. (**e**) 2D matrix flattening. (**f**) Morphological operations: closing and opening. (**g**) Morphological operation: skeletonization. (**h**) Information reprojection overlapped onto original point cloud (light green: skeleton, dark green: pipe start-end, red: elbow, black: connection, lines: pipe vectors).

Figure 8 showcases the described pipe information extraction process.

Finally, the valve and pipe information is refined. For the valves, its vector direction is recalculated taking into account the presence of pipes near the valve’s central point. If only one pipe is near, the valve vector is aligned to the pipe vector, if two or three pipes are present and two of them have parallel vectors, the valve pipe is aligned with that vector. Additionally, if three pipes are found near its central point, the valve type is set automatically to 3-way. For the pipes, vectors belonging to pipes of different instances that are near and parallel, are unified.

**Figure 9:**
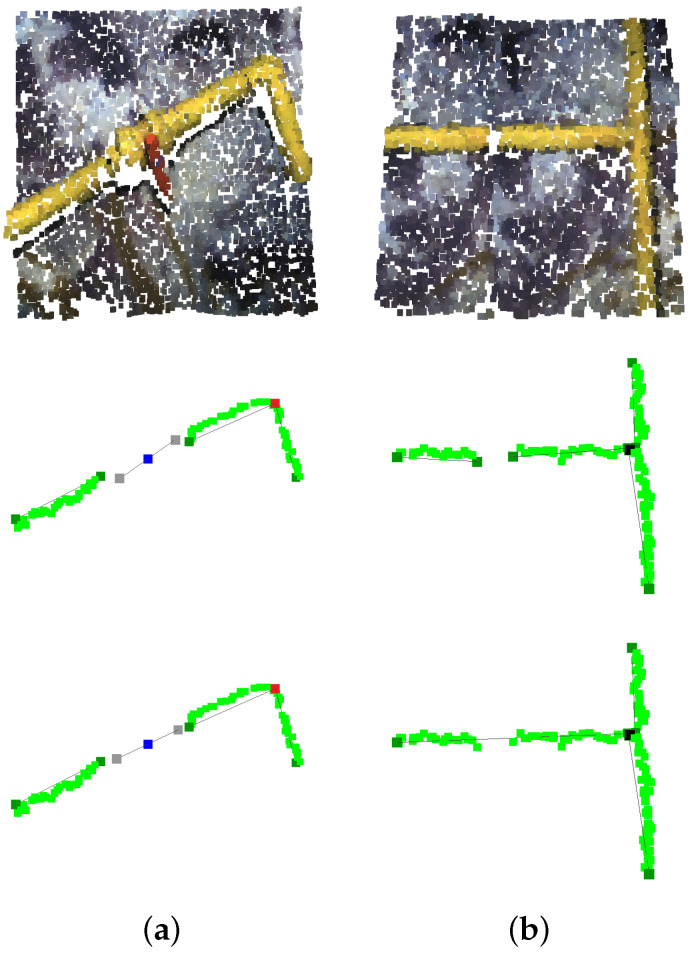
Information refinement: (**a**) Valve vector reorientation; (**b**) Pipe vector unification. Top: Original point cloud. Middle: information before refinement. Bottom: information after refinement.

Figure 9 shows examples of valve and pipe refinement.

#### 3.3.2. Information Unification Algorithm

This algorithm is built on top of the information provided by the previously detailed IEA and its end is to generate unified information maps by merging information from different point clouds. It is strictly necessary that the point clouds are referenced to a localised frame, whether it is an absolute frame such as geolocalisation or a relative one such as odometry.

Different methods are used to merge the pipe and valve information from new upcoming point clouds to the one already present in the information map. For the pipes, the algorithm checks if upcoming pipe chains are near chains present in the information map. Near chains are merged and new vectors and elbows are computed as explained in the IEA description. Moreover, a validation count is assigned to each pipe chain, indicating the number of times it has appeared in different point cloud information extractions, merged chains add up their validation counts. This validation count is used further into the algorithm to decide whether or not detection is a spurious false positive or a certain true positive.

For the connection points, the algorithm checks if new upcoming connections are situated near prior registered connections. Near connections are merged, averaging their 3D position. A validation count is also assigned to each connection point.

For the valves, the procedure is the same as for the connection points, with the addition that the valve vector direction is also averaged between the prior valve vector and the upcoming one.

Finally, for each *K* processed point cloud, the algorithm runs a validation count check, where pipes, connection points and valves with lower validation count than a determined threshold are discarded.

**Figure 10:**
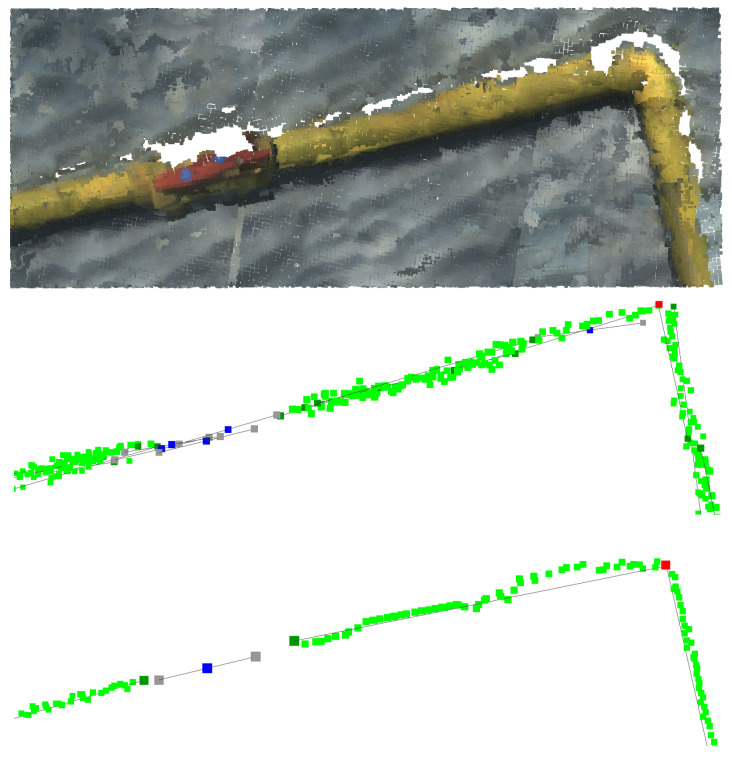
IUA implementation. **Top**: Overlapped point clouds. **Middle**: Overlapped extracted information from IEA. **Bottom**: Unified information from IUA.

Figure 10 presents the IUA output when implemented over a series of point clouds containing two pipes, a valve and an elbow. It can be seen how a false positive valve appears on the original information extracted near the elbow, but it is later eliminated by the algorithm count check as it is not found in any other point cloud information.

**Figure 11:**
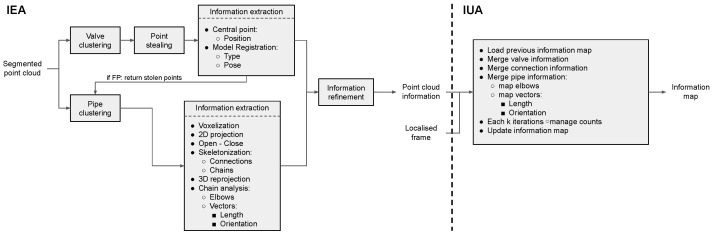
IEA and IUA algorithms flowchart, describing their workflow and interrelation.

Figure 11 presents a flowchart of both IEA and IUA algorithms, describing their workflow and interrelation. Additionally, the commented code of both algorithm implementations is provided in [46], which offers a deeper insight into the diverse algorithm steps and their numerous parameters that can be tweaked.

### 3.4. Validation and Evaluation Metrics

DGCNN is a highly efficient and effective network, obtaining great metrics in both object classification and segmentation tasks in indoor and outdoor scenarios [47]. However, it has never been tested and validated in underwater scenarios.

In order to validate the DGCNN, we use the 10k-fold cross-validation method [52]. With it, the train-validation partition of our dataset (see Figure 4) is split into ten equally sized subsets. Next, the network is trained ten times, each one using a different subset as validation and the nine remaining as training, generating ten models, which are then tested against the *T_BASE_* and *T_EXTRA_* test sets. The final performance is computed as the average results for the ten models. This method reduces the variability of the results, making them less dependent on the selected training and validation data, and therefore obtaining a more accurate performance estimation.

To evaluate a model performance, we make a point-wise comparison between the network predictions and their corresponding ground truth annotations. For each class, the number of correctly segmented points, True Positives (*TP*); and the number of incorrectly segmented points, False Positives (*FP*) or False Negatives (*FN*) is computed. The number of *TP*, *FP* and *FN* are used to calculate the *Precision*, *Recall* and *F1-score* for each class, following Equations (1)–(3).

(1) $$Precision=\frac{\mathrm{TP}}{\mathrm{TP}+\mathrm{FP}}\;,$$

(2) $$Recall=\frac{\mathrm{TP}}{\mathrm{TP}+\mathrm{FN}}\;,$$

(3) $$F1\text{-}score=2\cdot \frac{Recall\cdot Precision}{Recall+Precision}\;.$$

The goal of this work is not to work on a pixel-level segmentation, but to group pixels into instances from which to extract information. Therefore, it makes sense to evaluate the network performance on an instance level as well. In order to achieve that, we apply the clustering method explained in Section 3.3.2 to the network segmentation output and ground truth annotations. From there, we make use of the *Intersection over Union* (IoU) metric, which provides the similarity between two instances. The IoU value between two instances is calculated following Equation (4).

(4) $$\mathrm{IoU}=\frac{inst1\cap inst2}{inst1\cup inst2}=\frac{P_{shared}}{P_{inst1}+P_{inst2}-P_{shared}}\;.$$

where $P_{inst1/2}$ denotes the number of points forming instance one or two and $P_{shared}$ the number of points shared by both instances.

To determine whether a predicted instance is a *TP* or a *FP*, an IoU threshold value needs to be established. Following the criteria applied in the PASCAL VOC challenge [53], we set this threshold at *thr_iou_ = 0.5*. A predicted instance is classified as *TP* if the IoU value with any ground truth instance is greater than the *thr_iou_* and the prediction class ($C_{pred}$) is the same as the ground truth instance class ($C_{gt}$). Otherwise, the predicted instance is classified as a *FP* (Equation (5)).

(5) $$Inst.=\left\{\begin{array}{cc} \mathrm{TP}, & \mathrm{if}\;\mathrm{IoU}>=thr_{iou}\;\&\;C_{pred}==C_{gt}\;,\\ \mathrm{FP}, & \mathrm{otherwise}\;. \end{array}\right.$$

Ground truth instances that do not have an IoU > *thr_iou_* with any predicted instance are counted as undetected instances, *FN*.

Once each prediction instance is classified as either *TP* or *FP*, and the number of *FN* is obtained, the instance-level *Precision*, *Recall* and *F1-score* metrics are computed following the previous Equations (1)–(3).

Finally, to evaluate the information provided by the IEA and IUA, information ground truths are built, manually, over the *T_BASE_*, *T_EXTRA_* and *T_MAP_* test sets point cloud network segmentations, annotating the same information generated by the IEA for its comparison. The extracted information is compared to the ground truth annotations using different metrics for each type of information.

For the pipe information, the vectors are compared in terms of magnitude and direction, for the elbows and connections points, the distance difference from their ground truth annotation counterparts is measured.

For the valve information, the describing vector is compared only in terms of direction, since its magnitude is fixed by parameter, central valve point diversion is also measured. Lastly, the correct valve type classification is checked.

## 4. Experimental Results and Discussion

This section reports the results obtained by the DGCNN segmentation at pixel and instance levels. The IEA and IUA are also evaluated, checking the validity of the provided information.

### 4.1. DGCNN Segmentation Results

The results presented in this section are the average values obtained from the ten models generated when using the 10k-fold cross-validation method previously explained in Section 3.4.

Table 1 presents the pixel-level metrics when evaluating the DGCNN over the *T_BASE_* test set. The presented metrics are the per-class *F1-score (F1)* and its mean value *(mF1)*. Additionally, since the background class is not converted into instances nor used by the IEA or IUA, it makes sense to only focus on the pipe and valve classes when conducting the per-pixel evaluation of the network. The *2mF1* metric measures the mean *F1-score* only taking into account those two classes.

The DGCNN reaches a *F1-score* value of 92.4% for the pipe class, an 84.9% for the less represented and harder to identify valve class and a 99.7% for the prevailing background class, resulting in a mean *F1-score* of 92.3%. When only taking into account the pipe and valve classes, the network scores an *2mF1* of 88.7%.

Table 2 presents the instance-level metrics when evaluating the DGCNN over the *T_BASE_* test set. The presented metrics are the per-class Intersection over Union (*IoU*) and its mean value (*mIoU*) along the per-class *F1-score (F1)* and its mean value *(mF1)*.

The achieved *mIoU* value is 80.4%, which indicates a high overlap between predicted and ground truth instances. This similarity is reflected in the *mF1* score, reaching a value of 95.1%. The reached instance-level *mF1* score is higher than the obtained pixel-level *2mF1* score, this indicates that the applied pixel clustering allows us to match predicted and ground truth instances even when there exist pixel differences between them.

Table 3 and Table 4 present the pixel-level and instance-level metrics, respectively, when evaluating the DGCNN over the *T_EXTRA_* test set.

The results for the *T_EXTRA_* test set at both pixel-level and instance-level evaluations are equally good as the ones obtained for the *T_BASE_* test set, reaching a pixel-level *2mF1* of 87.2% and an instance-level *mF1* of 95.4%. This means that the DGCNN is able to generalise its training and avoid overfitting, being able to correctly segment more challenging point clouds with unseen pipe and valve connections and environment conditions like the ones present in the *T_EXTRA_* test set.

The metrics presented in this section outperform other state-of-the-art methods for pipe recognition: [35]-traditional computer vision algorithms over 2D underwater images achieving an F1-score of 94.1%, [7]-traditional computer vision algorithms over 2D underwater images achieving a mean F1-score over three datasets of 88.0% and [45]-deep learning approach for 2D drone imagery achieving a pixel-wise accuracy of 73.1%.

For the valve recognition, the only works that consider this class are [20,21], which use Bayesian techniques to perform 3D object recognition and classification of different types of valves and structural elements such as elbows or ’T’ junctions. Vallicrosa et al. [21] report an average F1-score of 78% in the object recognition and classification task, this metric is not directly comparable to our results since we do not distinguish between different types of valves and structural elements such as elbows or ’T’ junctions are extracted from pipe information, not identified as elements per se.

An issue of Vallicrosa et al. [21] work is the fact that one of the first steps of the proposed algorithm is to perform a normal estimation and region growing grouping over the analysed point cloud and classify each region into two groups, flat areas or points belonging to the pipeline structure. This method for selecting the pipeline structure points would produce critical failures in real-world implementations, as any non-flat area would be analysed as part of the pipeline system.

### 4.2. IEA Results

Table 5 shows the evaluation metrics obtained by the IEA when applied over the network segmentation of the *T_BASE_* and *T_EXTRA_* test sets altogether.

The IEA was able to detect all pipes, elbows, connections and valves present in the network segmentation, without generating any false positives.

For the pipe information, the vector magnitude (pipe length) and direction (pipe orientation) are evaluated. The difference between IEA output and ground truth for the magnitude is 1.94 cm, and $4.2^{\circ }$ for the direction. For the elbow and connection information, only the central point position is evaluated, for which the differences are 2.7 cm and 1.69 cm, respectively. With these metrics, it can be determined that the IEA is able to determine pipe length, orientation and its different structural elements with high accuracy.

For the valve information, the central point (valve position) and its vector direction (valve orientation) are evaluated, obtaining a divergence of 1.63 cm and $13.31^{\circ }$, respectively. Furthermore, the valve type is correctly identified 73.6% of the time. Even though the valve position is detected with high accuracy, there exists a small error when determining its orientation and a higher one when classifying its type.

**Figure 12:**
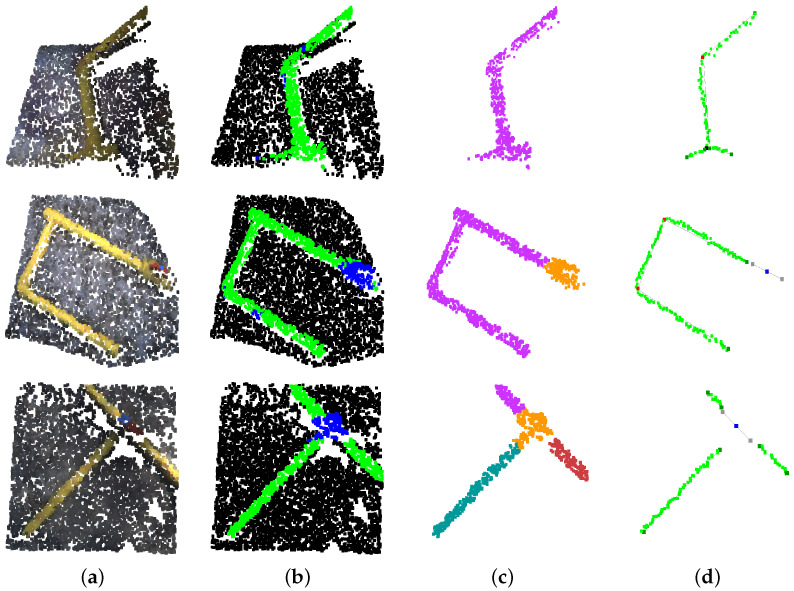
Neural network segmentation and IEA qualitative results. (**a**) Original point cloud. (**b**) Deep neural network segmentation. (**c**) Instance clustering. (**d**) Information extracted from IEA.

Qualitative results of the neural network segmentation and IEA output over diverse point clouds are shown in Figure 12.

### 4.3. IUA Results

Table 6 shows the evaluation metrics obtained after applying the IUA on the information extracted by the IEA from the network segmentation of the *T_MAP_* test set. For this execution, the validation count check was executed for five analysed point cloud information (K=5) with a count threshold of 2.

All pipes, elbows and valves were detected without generating any false positives. Additionally, the presented metrics are very similar to the ones obtained by the IEA execution on the *T_BASE_* and *T_EXTRA_* test sets, which implies that the IUA is able to merge the information from diverse point clouds while preserving its quality.

**Figure 13:**
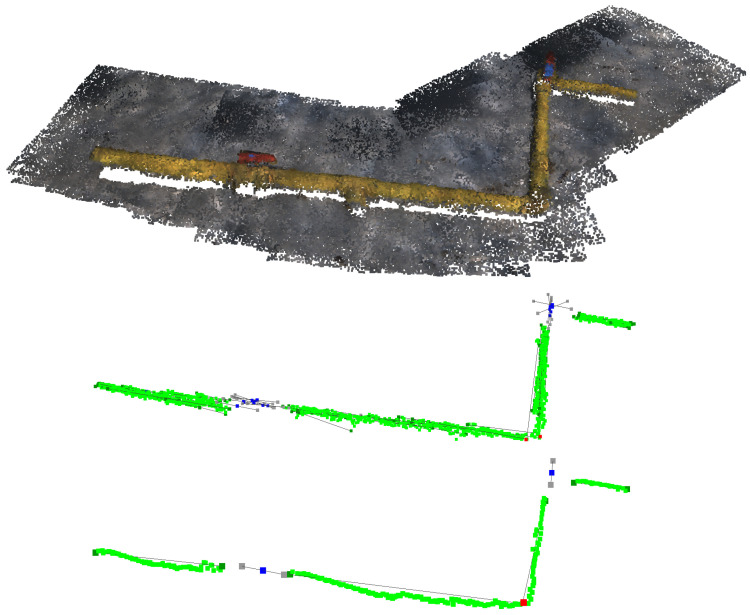
IUA implementation over *T_MAP_* test set. **Top**: Overlapped point clouds. **Middle**: Overlapped extracted information from IEA. **Bottom**: Unified information from IUA.

Figure 13 shows the *T_MAP_* test set original point clouds along the IEA information extraction output and the final unified information by the IUA.

## 5. AUV Online Implementation

An objective of this work is to implement the semantic segmentation network and information algorithms on an AUV and execute them online during manipulation and inspection tasks. This section describes the used AUV characteristics, the online implementation of the neural network and information algorithms, and its validation.

### 5.1. AUV Description

**Figure 14:**
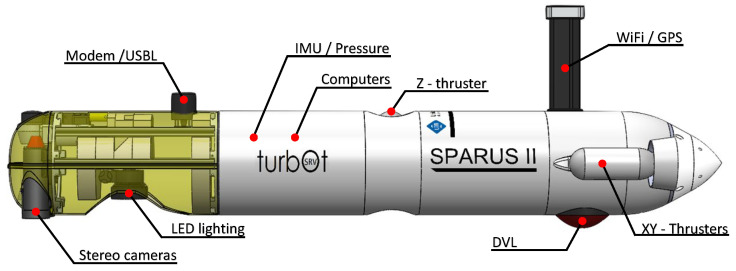
SPARUS II AUV.

The used AUV is a SPARUS II model unit [54] (Figure 14) equipped with three motors, granting it three degrees of mobility (surge, heave and yaw). Its navigation payload is composed of: (1) a Doppler Velocity Logger (DVL) to obtain linear and angular speeds and altitude; (2) a pressure sensor which provides depth measurements; (3) an Inertial Measurement Unit (IMU) to measure accelerations and angular speeds; (4) a Compass for heading; (5) a GPS to be georeferenced during surface navigation; and (6) a Short Baseline acoustic Link (USBL) used for localisation and data exchange between the robot and a remote station. Additionally, it is equipped with a stereo pair of Manta G283 cameras facing downwards.

The robot has two computers. One is dedicated to receiving and managing the navigation sensor data and running the main robot architecture developed under ROS (Intel i7 processor at 2.2 GHz, Intel HD Graphics 3000 engine and 4 GB of RAM). The second computer is used to capture the images from the stereo cameras and execute the online semantic segmentation and information algorithms (Intel i7 processor at 2.5 GHz, Intel Iris Graphics 6100 and 16 GB of RAM).

The localisation of the vehicle is obtained through the fusion of multiple state estimations produced by the DVL, IMU, Compass, GPS, USBL, visual odometry and a navigation filter [55]. This localisation can be integrated into the point clouds generated from the images captured by the stereo pair of cameras to spatially reference them, which is a requirement to execute the IUA.

### 5.2. Implementation

To perform the online implementation we design a pipeline based on ROS.

First, the images published by the stereo pair are transformed into point clouds to be processed by the neural network. To do so, diverse C++ ROS nodes are set up to: (1) rectify the raw images using the camera calibration parameters; (2) decimate the rectified images from their original size (1920×1440 pixels) to 960×720 pixels; (3) calculate the disparity map and generate the point clouds; and (4) downsample the point clouds using a voxel grid. Additionally, a python ROS node is set up to subscribe to the downsampled point clouds.

Following this, the point cloud is fed into a previously loaded inference graph of a DGCNN trained model, performing the semantic segmentation. From there, the IEA and IUA are executed. Finally, a publishing python ROS node is set up to publish the extracted information back into ROS to be accessed by other robots, sensors or actuators.

This pipeline achieves the implementation of the semantic segmentation network and information algorithms on an AUV and allows its execution online during manipulation and inspection tasks.

### 5.3. Validation

To validate the online execution, the frame rate of the output information stream is evaluated. An online execution was performed during the immersions conforming the *S_POOL-2_* and *S_SEA-3_* sets. In total, the online workflow was tested for 15′23″. For each execution the achieved output information stream frame rate and the time that each online execution step described in Section 5.2 takes are calculated.

For each immersion, the inspected pipe and valve configuration are different, making the IEA and IUA algorithms execution time vary, as the number and shape of pipes and valves are different, making the time analysis more robust as it covers a wider variety of scenarios.

**Figure 15:**
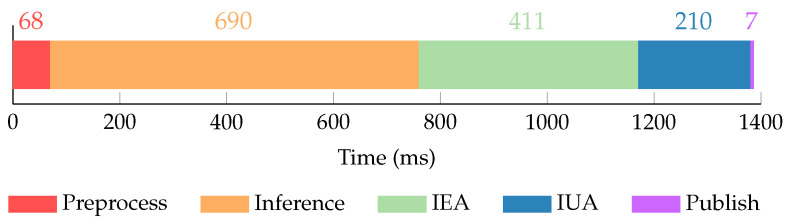
Online execution time breakdown.

The average output information stream frame rate and times for each online execution step are calculated as the mean value from both executions. Figure 15 presents a breakdown of the total average online execution time into its different steps.

The total average online execution time is 1.39 s, which results in an output information stream frame rate of 0.72 fps. The preprocessing step takes a mean of 68 ms (4.9% of the total time) and includes all operations to transform the images published from the stereo pair into point clouds to be processed by the neural network. The network inference takes the biggest amount of time with a mean of 690 ms (49.8% of the total time). Following, the IEA and IUA take a mean of 411 ms and 210 ms, accounting for 29.7% and 15.1% of the total time, respectively. Finally, the information publication takes a mean of 7 ms (0.5% of the total time).

The achieved output information stream frame rate is more than enough to perform manipulation tasks, as these kinds of operations in underwater scenarios tend to have slow and controlled dynamics. Additionally, for most manipulation tasks the IUA may not be executed, lowering the overall online execution time to 1.18 s, and thus increasing the achieved frame rate to 0.85 fps.

Regarding inspection tasks, a method to validate the achieved output information stream frame rate is to check if exists an overlap between the analysed point clouds, ensuring full coverage of the inspected area. To do so, the overlap between the original images from analysed point clouds is checked. This overlap depends on the camera displacement between the images from two consecutive analysed point clouds ($d_{KF}$) and on the height of the image footprint ($h_{FP}$). Then, the *overlap* can be expressed as:

(6) $$overlap=\left(h_{FP}-d_{KF}\right)\cdot h_{FP}^{-1}\;,$$

(7) $$d_{KF}=v\cdot frame\_rate^{-1}\;,$$

(8) $$h_{FP}=\left(a\cdot h_{image}\right)\cdot f^{-1}\;.$$

where *v* denotes the AUV velocity, *a* the navigation altitude, $h_{image}$ the image height pixels and *f* the camera focal length.

During inspection tasks, an AUV such as the SPARUS II can achieve velocities up to v=0.4 m/s and navigate at a minimum altitude of a=1.5 m. Using these parameters along the Manta G283 camera intrinsic focal length of f=1505.5p and image height resolution of $h_{image}=1440p$, the obtained overlap is 61.4%. Thus, the output information stream frame rate is high enough to get point clouds to overlap even when the AUV navigates at its maximum speed and minimum altitude.

## 6. Conclusions and Future Work

This paper presented the implementation of the DGCNN deep neural network to perform pixel-wise 3D segmentation of underwater pipes and valves from point clouds. To train the network, multiple immersions were conducted with an AUV to gather point clouds containing diverse pipe and valve configurations.

Two information algorithms were developed, the first one groups the segmented pixels into instances and implements object detection, detecting pipe and valve instances in a point cloud. Following this, it extracts information from the detected instances providing pipe vectors, gripping points, structural elements such as elbows or connections, and valve type and orientation. The second algorithm unifies information from spatially referenced point clouds, forming an information map of an inspected area.

Lastly, an ROS pipeline was built to execute the 3D segmentation and information extraction and unification algorithms online on an AUV, performing real-time underwater pipe and valve recognition, characterisation and mapping for inspection and manipulation tasks.

The neural network evaluation presented good results, reaching a mean *F1-score* value of 88.0% between the two conducted tests at a pixel-wise level and of 95.3% at an instance-level. Validating the use of the DGCNN deep neural network on underwater scenarios.

The information extraction algorithm results showcased excellent metrics when extracting information from pipe instances and their structural elements (elbows and connections), and good metrics when extracting valves position, orientation and type. The mapping algorithm was able to merge information from diverse point clouds, preserving its quality and deleting spurious false positive detections. This information can be used as first-stage inspection tools to provide layout maps of pipelines, or even directly detect pipe ruptures or deviations.

Finally, the online execution validation demonstrated that the output information stream frame rate is high enough to perform manipulation tasks and to get point clouds to overlap, permitting an adequate implementation of the information unification algorithm and ensuring full coverage of an inspected area. Additionally, the online implementation enables the use of real-time information to perform pipeline tracking and following during inspection tasks.

It is important to point out that the whole workflow presented in this work is executed using a simple point cloud as an input, no matter what its source is (i.e., stereo vision, sonar, laser). Thus, it can be implemented and utilised in multiple scenarios covering a wide range of applications.

Further developments will focus on studying the implementation of new deep neural networks to improve their segmentation performance and reduce the inference time, as this is the most time-consuming step of the online execution. Additionally, new ways of extracting pipe and valve information will be studied to improve pose and type detection using Bayesian techniques as presented by Vallicrosa et al. in [21], maybe even being able to detect the valve handle position, provide the valve state and allow the generation of pipeline flow diagrams.

## Figures and Tables

**Table 1:** DGCNN pixel-level metrics on *T_BASE_* test set.

*Class*	*F1 (%)*	*mF1 (%)*	*2mF1 (%)*
***Pipe***	92.4		
***Valve***	84.9	92.3	**88.7**
***Background***	99.7		

**Table 2:** DGCNN instance-level metrics on *T_BASE_* test set.

*Class*	*IoU (%)*	*mIoU (%)*	*F1 (%)*	*mF1 (%)*
***Pipe***	83.4	80.4	96.7	**95.1**
***Valve***	77.4		93.5	

**Table 3:** DGCNN pixel-level metrics on *T_EXTRA_* test set.

*Class*	*F1 (%)*	*mF1 (%)*	*2mF1 (%)*
***Pipe***	91.4		
***Valve***	83.0	91.4	**87.2**
***Background***	99.7		

**Table 4:** DGCNN instance-level metrics on *T_EXTRA_* test set.

*Class*	*IoU (%)*	*mIoU (%)*	*F1 (%)*	*mF1 (%)*
***Pipe***	82.7	79.7	96.3	**95.4**
***Valve***	76.8		94.6	

**Table 5:** IEA metrics on *T_BASE_* and *T_EXTRA_* test sets.

*Info.*	Δ*C_Point_* (cm)	Δ*V_Magnitude_* (cm)	Δ*V_Direction_* (${}^{\circ }$)
***Pipe***	-	1.94	4.20
***Elbow***	2.70	-	-
***Conn.***	1.69	-	-
***Valve***	1.63	-	13.31

**Table 6:** IUA metrics on *T_MAP_* test set.

*Info.*	Δ*C_Point_* (cm)	Δ*V_Magnitude_* (cm)	Δ*V_Direction_* (${}^{\circ }$)
***Pipe***	-	3.12	2.42
***Elbow***	3.40	-	-
***Conn.***	-	-	-
***Valve***	3.59	-	13.92

## Data Availability

The data presented in this study are openly available in Kaggle with DOI:10.34740/kaggle/dsv/2759939.
